# IL-1β/IL-6/CRP and IL-18/ferritin: Distinct Inflammatory Programs in Infections

**DOI:** 10.1371/journal.ppat.1005973

**Published:** 2016-12-15

**Authors:** Jeroen Slaats, Jaap ten Oever, Frank L. van de Veerdonk, Mihai G. Netea

**Affiliations:** Department of Internal Medicine and Radboud Center for Infectious Diseases, Radboud University Medical Center, Nijmegen, The Netherlands; Stony Brook University, UNITED STATES

## Abstract

The host inflammatory response against infections is characterized by the release of pro-inflammatory cytokines and acute-phase proteins, driving both innate and adaptive arms of the immune response. Distinct patterns of circulating cytokines and acute-phase responses have proven indispensable for guiding the diagnosis and management of infectious diseases. This review discusses the profiles of acute-phase proteins and circulating cytokines encountered in viral and bacterial infections. We also propose a model in which the inflammatory response to viral (IL-18/ferritin) and bacterial (IL-6/CRP) infections presents with specific plasma patterns of immune biomarkers.

## The Inflammatory Response

Inflammation is a protective response by the body that aims to remove invading pathogens, neutralize noxious stimuli, and initiate tissue repair. Inflammation is triggered when innate immune cells sense evolutionary conserved structures on pathogens (pathogen-associated molecular patterns [PAMPs]) or endogenous stress signals (damage-associated molecular patterns [DAMPs]) through germline-encoded pattern recognition receptors (PRRs). PRRs are mainly expressed by macrophages and dendritic cells, although they have also shown to be expressed by other immune and non-immune cells including neutrophils, lymphocytes, fibroblasts, and epithelial cells [1]. During an infection, cell stress provoked by invading pathogens also leads to the release of DAMPs that synergize with PAMPs to activate PRRs. The role of DAMPs in PRR activation is particularly pronounced during viral infections as viral spread depends on the fate of infected cells.

**Fig 1:**
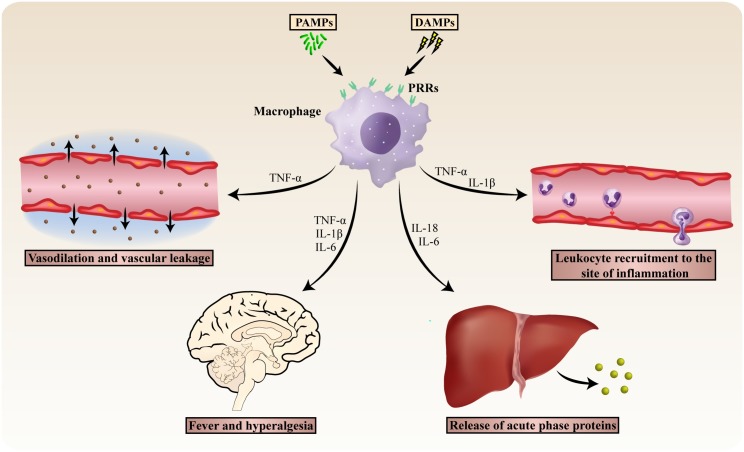
The acute inflammatory response mediated by the release of pro-inflammatory cytokines. Following PAMP or DAMP recognition, PRRs trigger proinflammatory and antimicrobial responses by inducing the release of a broad range of cytokines. The archetypical pro-inflammatory cytokines TNF-α, IL-1β, and IL-6 are rapidly released upon PRR activation, and they all act as endogenous pyrogens by increasing the hypothalamic thermoregulatory set-point [82]. In addition, TNF-α and IL-1β orchestrate the release of chemokines and expression of leukocyte adhesion molecules on vascular endothelium, promoting the rapid and efficient recruitment of leukocytes towards inflammatory foci [83–85]. TNF-α is also responsible for multiple hallmark signs of inflammation by inducing local vasodilation (rubor, calor) and vascular leakage (causing swelling) [86, 87]. Furthermore, IL-1β evokes inflammatory hyperalgesia and is well known for its induction of IL-6 [88, 89]. IL-6, in turn, is a major inducer of acute-phase protein production by hepatocytes [90]. PAMP, pathogen-associated molecular pattern; DAMP, damage-associated molecular pattern; PRR, pattern recognition receptor.

PRR activation triggers a complex array of inflammatory processes through the release of proinflammatory cytokines, with the induction of acute phase proteins (APPs) being a prominent feature of the inflammatory cascade (Fig 1). APPs comprise a homeostasis-restoring class of proteins whose plasma concentration increases in response to inflammatory insults. Plasma inflammatory cytokines and APPs have been established as a valuable tool in the diagnosis, management, and prognosis of inflammatory diseases, given their exceptional sensitivity for systemic inflammation [2]. Because inflammatory cytokines and APPs are highly heterogeneous with a wide variety of biological functions, we asked ourselves whether we could discriminate between different types of inflammatory reactions based on distinct cytokine/APP profiles. This review discusses APPs and major cytokines involved in viral- and bacterial-induced inflammation and proposes a novel model in which two types of inflammatory reactions present with differential plasma levels of immune biomarkers. We also aim to propose a pathophysiological distinction between the induction of the acute phase response in bacterial versus viral infection.

## Plasma Cytokine Markers of Inflammation

Plasma cytokine profiling is routinely used in patients with inflammation to define the pathophysiological phenotype, thereby playing a pivotal role in the diagnosis and therapeutic decision-making. The pro-inflammatory cytokines IL-1α, IL-1β, and IL-18 are inflammatory plasma markers belonging to the IL-1 family of cytokines, which are all synthesized as precursor proteins. The IL-1α precursor (pro-IL-1α) is biologically active and can be found constitutively inside cells under homeostatic conditions [3]. Pro-IL-1α is passively released from dead, dying, or injured non-apoptotic cells and acts as a major DAMP capable of triggering powerful inflammatory responses [4]. IL-1β and IL-18 are mainly produced by monocytes/macrophages in response to PAMP/DAMP recognition by PRRs [5]. Unlike pro-IL-1α, the precursors of IL-1β and IL-18 (pro-IL-1β and pro-IL-18) are biologically inactive and require proteolytic cleavage into biologically active mature cytokines. This proteolytic cleavage mostly depends on caspase-1 activation through the formation of multimeric protein complexes termed inflammasomes. Hence, a two-step model has been proposed: first, activation of PRRs on host cells induces transcription of pro-IL-1β and pro-IL-18; second, activation of the inflammasome by PAMPs or DAMPs results in the posttranslational cleavage of the pro-cytokines into mature IL-1β and IL-18 [5].

Although the release of IL-1β and IL-18 involves similar processes, their functions differ considerably. In synergism with IL-12, IL-18 acts as bridge to link the innate immune response to IFN-γ production by driving T-helper (Th) 1 polarization and priming NK cells, both resulting in high-level production of IFN-γ [6, 7]. IFN-γ is crucial for early host defense against infections by stimulating the phagocytosis and intracellular killing of pathogens, particularly intracellular bacteria and fungi. IFN-γ also plays an important role in establishing an antiviral state for long-term control through the induction of key antiviral enzymes, most notably protein kinase R [8]. In addition, IL-18 harbors the unique property of inducing Fas ligand expression on NK cells, facilitating their killing of infected cells by Fas-mediated apoptosis [9].

In contrast to IL-18, IL-1β negatively regulates IFN-γ-mediated responses. IL-1β is a potent inducer of COX-2 expression, leading to the production of large amounts of prostaglandin E2 (PGE2). PGE2, in turn, directly acts on T cells to suppress IFN-γ production, thereby suppressing Th1 immunity and driving Th17 polarization [10]. Another downstream action of IL-1β involves upregulation of the pleotropic cytokine IL-6, which is a key factor in priming naïve T cells for Th17 differentiation [11]. IL-6 can also inhibit the immunosuppressive functions of regulatory T cells and prevent Th17 cells from converting into regulatory T cells, illustrating a pivotal role for IL-6 in shaping the adaptive immune response in favor of Th17 immunity [12, 13]. Th17 responses, which are characterized by the production and release of IL-17 and IL-22, are critical for epithelial and mucosal host defense against extracellular bacteria and fungi [14]. IL-17 and IL-22 cooperatively enhance mucosal barrier function by stimulating the expression of antimicrobial peptides and inducing neutrophil recruitment [14, 15]. Interestingly, Th17 responses may contribute to a suboptimal host defense against viruses by upregulation of anti-apoptotic molecules, thereby blocking target cell destruction by cytotoxic T cells and enhancing the survival of virus-infected cells [16]. Thus, although Th17 responses are very efficient in orchestrating the clearance of extracellular bacteria and fungi, the killing of intracellular pathogens such as intracellular bacteria and viruses may be more effective in the setting of a strong Th1 response.

## Acute-Phase Proteins

To assess the presence of inflammation in a clinical setting, laboratories routinely assess the plasma concentrations of various APPs as robust biomarkers of inflammation. APPs are produced primarily by hepatocytes in response to various inflammatory cytokines, most notably IL-1β and IL-6, although IL-18 has also been implicated in APP release [17]. C-reactive protein (CRP) is the prototype of human APPs. In healthy individuals, CRP is found in trace amounts with a median plasma concentration of 0.8 mg/L, while CRP values rise sharply up to 1,000-fold after an inflammatory stimulus [18]. CRP remains stable over prolonged time periods and has a half-life of 19–20 hours [19]. Because this half-life remains constant under conditions of health and disease, the sole determinant of circulating CRP is its synthesis rate, which directly reflects the intensity of the inflammatory process [19]. This makes CRP a powerful marker for disease activity in infectious and inflammatory diseases.

CRP plays an important role in innate immunity as early defense mechanism against infections. After binding to microbial polysaccharides or ligands exposed on damaged cells, CRP can directly mediate their phagocytosis by engaging with Fc-receptors on phagocytic cells [20, 21]. Ligand-bound CRP can also mediate phagocytosis indirectly by activation of the classical complement pathway through interaction with C1q [22]. This activation process results in C3/C4 opsonization of pathogens and apoptotic cells, enhancing complement receptor-mediated phagocytosis. However, CRP attenuates the formation of a downstream membrane attack complex on the surface of invading microbes or damaged cells through recruitment of factor H, thereby protecting the cells from lysis [23]. Thus, activation of the complement cascade by CRP limits the inflammatory response by promoting opsonization while avoiding the pro-inflammatory effects of cell lysis.

Another inflammatory plasma marker that has been extensively used in clinical practice is the APP ferritin. Ferritin is a ubiquitous intracellular iron storage protein composed of 24 light (L) and heavy (H) ferritin monomers. By storing iron in a non-toxic form, ferritin prevents iron from catalyzing radical formation through Haber-Weiss or Fenton chemistry. During infection and inflammation, iron is withdrawn from the circulation and is redirected to hepatocytes and macrophages, thereby reducing the availability of this essential nutrient to invading pathogens [24]. The resulting iron overload in hepatocytes and macrophages enhances the translation of ferritin through the iron response protein [25]. Part of the elevated ferritin load in macrophages may translocate to the lysosomal compartment, where it protects this compartment from reactive iron, followed by ferritin secretion through the secretory-lysosomal pathway [26]. Ferritin may also enter the circulation via the classical ER/Golgi-dependent secretory pathway in hepatocytes [27, 28]. Another possible mechanism for ferritin secretion involves leakage from damaged cells, explaining the firm association between serum ferritin and markers of hepatocellular damage [29]. It is, however, important to note that circulating ferritin lacks most of the iron it contained when being intracellular.

Although many aspects of the fundamental biology of serum ferritin remain surprisingly unclear, various immune regulatory roles have been attributed to extracellular ferritin. Unlike L-ferritin, H-ferritin modulates the immune response by suppressing lymphocyte blastogenesis and myelopoiesis, possibly through inhibition of transferrin-mediated iron uptake, as iron is required for cell proliferation and differentiation [30–32]. H-ferritin also downregulates the immune response through induction of the anti-inflammatory cytokine IL-10 by regulatory T cells [33]. In addition, H-ferritin physically interacts with the chemokine receptor CXCR4, thereby attenuating CXCR4-mediated leukocyte migration to inflammatory sites [34]. It remains, however, paradoxical that circulating ferritin predominantly consists of L subunits, whereas most evidence supports immune modulatory functions for H-ferritin.

Besides CRP and ferritin, various other APPs have been used in clinical practice. One such protein is serum amyloid A (SAA), whose plasma concentration can rapidly increase up to 1,000-fold in response to inflammatory stimuli [35]. SAA is mainly produced in the liver and serves as an innate recognition molecule that opsonizes gram-negative bacteria for phagocytosis [36]. SAA also induces powerful and rapid secretion of proinflammatory cytokines by monocytes and macrophages, thereby augmenting the early host response to invading pathogens [37, 38]. Another fast-responding APP that has been of particular interest is the prohormone procalcitonin. Procalcitonin is secreted by parafollicular C-cells of the thyroid gland under normal conditions but can be secreted by numerous other cell types throughout the body in response to proinflammatory stimulation, culminating in markedly elevated serum procalcitonin levels [39]. However, the exact physiological function of procalcitonin during the acute phase response remains obscure and requires further study.

## Plasma Inflammatory Profiles in Viral and Bacterial Infections

One of the most frequent challenges that physicians face in clinical practice is the difficulty of discriminating between viral and bacterial infections. Timely discrimination between viral and bacterial etiologies is not only required for appropriate treatment but can also prevent unnecessary morbidity and even mortality. A rapid and powerful tool that assists in the diagnosis of infectious diseases is the monitoring of host immune responses through circulating cytokines and APPs.

An interesting pattern of inflammatory plasma markers emerges in bacterial infections. Many bacterial diseases are characterized by elevated levels of circulating IL-1β and IL-6 with a concomitant increase in plasma CRP, explaining the good correlation between plasma levels of IL-6 and CRP [40]. It has been shown that circulating concentrations of both IL-6 and CRP are markedly higher in patients with community-acquired bacterial infections as compared to patients with viral infections [41]. In addition, plasma IL-6 and CRP concentrations are significantly more elevated in bacterial enterocolitis as compared to viral enterocolitis [42]. The combination of IL-6 and CRP plasma biomarkers can even be used to predict serious bacterial infections in young febrile infants [43]. Importantly, IL-6–induced CRP levels are able to distinguish between specific viral and bacterial etiologies that remain daunting challenges in clinical practice, including the discrimination between bacterial pneumonia and influenza infections as well as the discrimination between streptococcal pharyngitis and infectious mononucleosis [44].

Unlike many bacterial infections, viral infections are commonly characterized by elevated plasma levels of the pro-inflammatory cytokine IL-18, together with increased circulating ferritin concentrations. In healthy adults, IL-18 circulates in relatively low concentrations of less than 200 pg/mL, while circulating ferritin concentrations are usually in the range of 120 μg/L [45, 46]. However, during the acute stages of an Epstein-Barr virus (EBV) infection, plasma IL-18 concentrations can easily exceed 1,000 pg/mL, with median ferritin up to 431 μg/L [47]. IL-18 and ferritin are also strongly induced during chronic hepatitis B and C virus infections [48–51]. The human immunodeficiency virus (HIV) disease is another viral infection that has been characterized by increased circulating levels of both IL-18 and ferritin. During HIV infection, plasma IL-18 levels exceed 1,000 pg/mL [52, 53]. In addition, circulating ferritin in HIV patients ranges around a median of 487 μg/L and has been associated with HIV disease progression [54, 55]. The most striking elevation of circulating ferritin is seen in patients suffering from acute dengue infections, with median plasma ferritin levels up to 1,264 μg/L [56, 57]. Moreover, circulating levels of both IL-18 and ferritin show strong correlation with dengue disease severity and, therefore, may be considered as a tool to predict disease progression [57, 58]. It is, however, important to note that elevated levels of circulating IL-18 in dengue virus infections concur with increased levels of the antagonistic IL-18 binding protein (IL-18BP), resulting in unchanged plasma concentrations of free, biologically active IL-18 [59]. This demonstrates that special attention should be paid to circulating levels of free IL-18 molecules when studying the IL-18 response in infectious diseases, particularly because the ELISA kits for IL-18 detection measure the mature form of the cytokine, both free and complexed with IL-18BP. Besides its role in infectious diseases, IL-18 has been implicated in the pathophysiology of various other diseases, but this falls outside the scope of this review and has been reviewed extensively in earlier reviews (see [60]).

**Fig 2:**
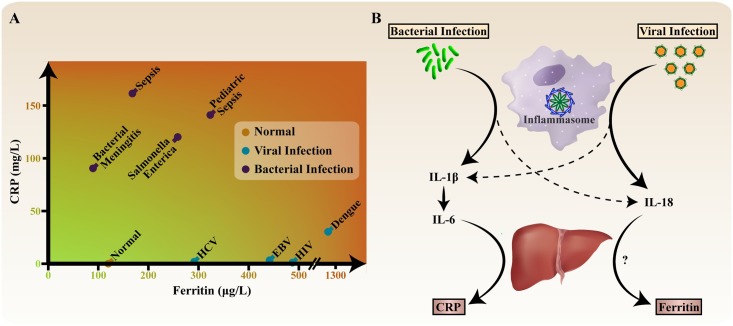
Bacterial- and viral-induced inflammation are characterized by differential plasma levels of CRP and ferritin. (**A**) Mean or median concentrations of circulating CRP and ferritin in various viral and bacterial infections illustrate that viral infections are generally characterized by high plasma ferritin with concomitant low circulating CRP [18, 45, 47, 54, 57, 91–94], while bacterial infections are commonly characterized by high plasma CRP levels [95–100]. (**B**) Proposed model in which the induction of IL-1β/IL-6 in response to bacterial infections contributes to elevated plasma levels of CRP, while viral infections are characterized by an IL-18 response, culminating in hyperferritinemia. Importantly, IL-1/IL-6/CRP and IL-18/ferritin do not fully reflect the bacterial-viral infection dichotomy, as various bacterial infections are known to elevate plasma IL-18 levels while some viral infections are known to raise plasma IL-1β levels [72, 73]. The direct correlation between circulating concentrations of IL-18 and ferritin has not yet been investigated and should be assessed in future studies. HCV: hepatitis C virus infection; EBV: Epstein-Barr virus infection; HIV: human immunodeficiency virus infection.

Although bacterial infections are commonly characterized by the induction of IL-6 and CRP, viral infections are generally associated with marked elevation in plasma IL-18 and ferritin with concomitant low circulating CRP levels (Fig 2A) [47]. Therefore, we propose a model in which bacterial- and viral-induced inflammatory responses present with differential plasma levels of CRP and ferritin (Fig 2B). Upon viral infection, IL-18 release induces a marked elevation of circulation ferritin, explaining the frequently observed hyperferritinemia in viral infections. IL-18 also stimulates Th1 immune responses, which play a crucial role in the host defense against intracellular microbes through the induction of IFN-γ. In contrast, bacterial infections are commonly associated with an extensive release of IL-1β, thereby stimulating the hepatocytic CRP secretion through the induction of IL-6. CRP, in turn, acts as innate weapon in early host defense by promoting the phagocytosis of bacteria. IL-1β also stimulates a Th17 immune response, which is crucial for epithelial and mucosal defense against extracellular bacteria. Thus, the IL-18 response in viral infections is responsible for hyperferritinemia, while bacterial infections are characterized by an IL-1β/IL-6 response, culminating in elevated plasma levels of CRP.

The host immune response to invading pathogens is orchestrated by a complex network of cytokines and acute phase reactants that are mainly represented by, but are not limited to, the IL-6/CRP and IL-18/ferritin axes described above. Differences in several other cytokine plasma markers and APPs have been described in bacterial and viral infections (Tables 1 and 2). Particular interest has been raised for SAA, which is significantly increased in patients with bacterial infections as compared to patients with viral infections [44]. This increase positively correlates with circulating CRP levels, and some authors have considered SAA to be equivalent to CRP in clinical practice, although SAA might be a more sensitive marker in infections with low inflammatory activity [44]. Procalcitonin has emerged as a promising biomarker in infectious inflammation. The diagnostic accuracy of procalcitonin has proven superior to both CRP and SAA in the early identification of bacterial infections, and procalcitonin serves as prognostic indicator for sepsis [61, 62]. The secretion of procalcitonin during these bacterial infections is stimulated by cytokine plasma markers such as IL-6 and tumor necrosis factor-α, whereas viral infections commonly attenuate the procalcitonin response, likely due to increased IFN-γ production [63, 64].

**Table 1:** Changes in plasma cytokine concentrations during infections.

	Bacterial Infection	Viral Infection
**IL-1α**	Low / N.D. [71]	N.D. [59]
**IL-1β**	Increased [71]	Increased [72]
**IL-1Rα**	Increased* [41]	Normal [41]
		N.D. [59]
**IL-2**	Increased* [41]	Increased [41]
**IL-6**	Increased* [41]	Low / ND [41, 59]
**IL-18**	Increased [73]	Increased [58, 59]
**IFN-α**	N.D. [74]	Increased† [59, 74]
**IFN-γ**	Increased [75, 76]	Increased [59]
**TNF-α**	Increased* [41, 71]	Normal [41]
		Increased [59, 72]

* Direct comparison between viral and bacterial infections revealed higher circulating levels in bacterial infections [41].

^†^ Direct comparison between viral and bacterial infections revealed higher circulating levels in viral infections [74]. N.D.: not detected.

**Table 2:** Changes in circulating concentrations of acute phase proteins during infections.

	Viral and Bacterial Infections
**CRP**	Stimulated by both viral and bacterial infections, but reaches higher values during bacterial infections [44, 77, 78]
**SAA**	
**Procalcitonin**	
**Ferritin**	Elevated in viral infections [47, 56]
**Retinol**	Decreased during infections [79]
**Haptogloblin**	Not significantly different between neonates with and without an infection [80]
**α1-antitrypsin**	
**LPS binding protein**	Elevated in bacterial infections as compared to viral infections [68]
**sTREM-1**	
**Neutrophil lipocalin**	More elevated in bacterial infections as compared to viral infections [81]

CRP: C-reactive protein; SAA: serum amyloid A; sTREM-1: soluble triggering receptor expressed on myeloid cells-1.

An interesting plasma inflammatory profile has been observed in macrophage activating syndrome (MAS). MAS comprises a heterogeneous group of life-threatening disorders featuring excessive activation of T cells and macrophages, leading to a cytokine storm. The development of MAS can be triggered by infectious diseases of viral or bacterial etiology and has been associated with exceptionally high serum levels of free IL-18 with concomitant hyperferritinemia, reflecting an IL-18/IL-18BP imbalance [65]. The circulating levels of IL-18 are remarkably high in both MAS and viral infections, even in comparison to severe bacterial sepsis. A likely explanation resides in the origin of circulating IL-18, with monocytes/macrophages being the main source of IL-18 in diseases of bacterial origin, while the largest amount of circulating IL-18 in MAS and viral diseases might originate from damaged endothelium. Anti-cytokine therapy using the IL-1 receptor antagonist anakinra provides a survival benefit for severe septic patients with features of MAS [66]. Because IL-1 has been shown to induce caspase-1 expression required for subsequent proteolytic maturation of pro-IL-18, anakinra can indirectly block the production of biologically active IL-18, thereby counteracting the overwhelming IL-18 response in septic patients with features of MAS [67].

## Conclusions and Future Perspectives

The model we propose describes the pathophysiology underlying the distinct immune responses to bacterial and viral infections, thereby offering new incentives for future research. However, our model does not serve as direct diagnostic tool in clinical practice, because considerable controversy exists about the diagnostic benefit of CRP, and combining CRP with other circulating biomarkers (e.g., IL-6, IL-18, and procalcitonin) was found not to improve the prediction of microbiological etiology in patients with lower respiratory tract infection [68, 69]. This emphasizes the importance of using inflammatory plasma markers as integral part of the diagnostic armamentarium, in which the clinical picture and patient’s history remain cornerstones. Further studies should elucidate the sensitivity and specificity of a plasma inflammatory signature consisting of IL-6, CRP, IL-18, and ferritin in distinguishing between viral and bacterial infections. It will also be important to assess the direct correlation between circulating concentrations of IL-18 and ferritin in viral infections, as current studies have only focused on either IL-18 or ferritin separately.

A large array of bacterial and viral infections should be investigated, as it may be expected that these two types of inflammatory reactions (IL-1/IL-6/CRP versus IL-18/ferritin) will not fully reflect the bacterial–viral infection dichotomy. In line with this, patients with influenza infection seem to have an inflammatory reaction more closely resembling a bacterial rather than viral infection [68], although this discrepancy might also be explained by an altered immune response due to (undetected) bacterial co-infection or the interference of influenza with caspase-1 activation [70]. More studies on various infections in larger cohorts are, thus, needed, and should also include fungal and parasitic infections.
